# Modeling of adult patient falls and the repercussions to Nursing as a second victim

**DOI:** 10.1590/1518-8345.5830.3618

**Published:** 2022-08-01

**Authors:** Deise Vacario de Quadros, Ana Maria Müller de Magalhães, Priscila Wachs, Isis Marques Severo, Juliana Petri Tavares, Daiane Dal Pai

**Affiliations:** 1 Hospital de Clínicas de Porto Alegre, Porto Alegre, RS, Brasil.; 2 Universidade Federal do Rio Grande do Sul, Escola de Enfermagem, Porto Alegre, RS, Brasil.; 3 Pontifícia Universidade Católica do Rio Grande do Sul, Porto Alegre, RS, Brasil.

**Keywords:** Nursing, Accidental Falls, Adverse Event, Occupational Health, Workflow, Patient Safety, Enfermagem, Acidentes por Quedas, Evento Adverso, Saúde do Trabalhador, Modelagem do Processo, Segurança do Paciente, Enfermería, Accidentes por Caídas, Evento Adverso, Salud Laboral, Flujo de Trabajo, Seguridad del Paciente

## Abstract

**Objective:**

analyze the falls of adult hospitalized patients and their repercussions on the Nursing worker as the second victim.

**Method:**

an exploratory, descriptive and qualitative study conducted in two stages - identification of falls with moderate to severe damage and the modeling of falls using the software Functional Resonance Analysis Method; and analysis of the repercussions on the worker as the second victim by means of semi-structured interviews submitted to Content Analysis, with 21 Nursing workers.

**Results:**

a total of 447 falls of adult patients were identified, 12 of which with moderate to severe damage, occurred in the absence of the companion, while using sleep inducing, hypotensive or muscle strength altering medications. The modeling identified 22 functions related to the Standard Operating Procedure, to the fall prevention protocol and to the post-event actions. Of these, eight presented variability in their execution. In the second step, the categories “The complexity of care to prevent falls” and “Feelings of the second victim” emerged.

**Conclusion:**

falls are complex phenomena and prevention requires involvement of the patient, family and multi-professional team. The professionals involved are second victims and experience feelings of guilt, fear, anguish and helplessness. This study can contribute to the multi-professional spirit and to the early approach to second victims.

## Resumo

**Objetivo:**

analisar as quedas de pacientes adultos hospitalizados e suas repercussões no trabalhador de Enfermagem como segunda vítima.

**Método:**

estudo exploratório, descritivo e qualitativo realizado em duas etapas - identificação das quedas com dano moderado ao grave e a modelagem das quedas com uso do *software Functional Resonance Analysis Method* e análise das repercussões no trabalhador como segunda vítima por meio de entrevistas semiestruturadas submetidas à Análise de Conteúdo, com 21 trabalhadores de Enfermagem.

**Resultados:**

identificadas 447 quedas de pacientes adultos, sendo 12 com dano moderado ao grave, ocorridas na ausência do acompanhante, no uso de medicamentos indutores do sono, hipotensores ou que alteram a força muscular. A modelagem identificou 22 funções relacionadas ao Procedimento Operacional Padrão, ao protocolo de prevenção de quedas e às ações pós-evento. Dessas, oito apresentaram variabilidade em sua execução. Na segunda etapa, emergiram as categorias “A complexidade dos cuidados para prevenir as quedas” e “Sentimentos da segunda vítima”.

**Conclusão:**

as quedas são fenômenos complexos e a prevenção exige envolvimento do paciente, família e equipe multiprofissional. Os profissionais envolvidos são segundas vítimas e experimentam sentimentos de culpa, medo, angústia e impotência. Este estudo pode contribuir com a multiprofissionalidade e na abordagem precoce às segundas vítimas.

## Resumen

**Objetivo:**

analizar las caídas de pacientes adultos hospitalizados y sus repercusiones en el trabajador de Enfermería como segunda víctima.

**Método:**

estudio exploratorio, descriptivo y cualitativo realizado en dos etapas - Identificación de las caídas con daño moderado a grave y el modelaje de las caídas utilizando el *software Functional Resonance Analysis Method* y Análisis de las repercusiones en el trabajador como segunda víctima por medio de entrevistas semiestructuradas posteriormente sometidas al Análisis de Contenido, en 21 trabajadores de Enfermería.

**Resultados:**

fueron identificadas 447 caídas de pacientes adultos, siendo 12 con daño moderado a grave, ocurridas en la ausencia del acompañante, al usar medicamentos inductores de sueño, hipotensores y/o los que alteran la fuerza muscular. El modelaje identificó 22 funciones relacionadas al Procedimiento Operacional Estándar, es decir el protocolo de prevención de caídas y las acciones seguidas después del evento. Entre estas, ocho presentaron variabilidad en su ejecución. En la segunda etapa, surgieron las categorías “La complejidad de los cuidados para prevenir las caídas” y “Sentimientos de la segunda víctima”.

**Conclusión:**

las caídas son fenómenos complejos y la prevención exige la participación del paciente, familia y equipo multiprofesional. Los profesionales involucrados son segundas víctimas y experimentan sentimientos de culpa, miedo, angustia e impotencia. Este estudio puede contribuir con la multiprofesionalidad y el abordaje precoz a las segundas víctimas.

**Highlights**

(1) The absence of a companion represents variability for the occurrence of the fall.

(2) Falls prevention involves the patient, the family and the multi-professional team.

(3) Nursing professionals involved in falls are second victims.

(4) Fear, guilt, anguish and helplessness are feelings experienced by the second victim.

(5) Process modeling is an effective strategy in fall prevention planning.

## Introduction

The identification of the increase in the number of Adverse Events (AE), by the World Health Organization (WHO), led to an international effort which saw, in patient safety, the great challenge to improve the quality of services and their management, proposing methodologies to reduce risks and offer quality care^1^. Among the events that compromise patient safety are falls, which present up to 70% of associated injuries and increased hospitalization time, resulting in 10% of deaths^2^.

The implementation of protocols and the reformulation of work processes to treat and prevent falls are a challenge within hospitals, dynamic environments, considered Complex Sociotechnical Systems (CSS)^3^. In contexts like these, Resilience Engineering seeks improvements that may imply in the final result and uses process modeling to emphasize safety and understand the prescribed work (that previously determined, sequence of actions described in protocols) and the real work (the actions effectively performed, in interaction between people and with the environment)^4^ ^-^ ^6^.

The Functional Resonance Analysis Method (FRAM) was proposed from the perspective of Resilience Engineering and is used for modeling CSS^4^ ^-^ ^6^. FRAM presents, visually, its functions and the interaction between them, thus evidencing the complexity with the different elements that interact in a non-linear way^4^ ^,^ ^6^. FRAM considers, as a function, what needs to be performed to achieve a certain goal or process under analysis. Each function can be described considering its input (what activates the function), output (the result of the function), time (temporal aspects for the function to be executed), control (supervises or regulates the function), precondition (necessary condition for the execution of the function) and resources (necessary or consumed during the function)^4^. FRAM modeling can be retrospective, to gain insights and reflections on events that have already occurred such as incidents or accidents or prospective, to identify and manage risks or understand the behavior of the system^5^.

Besides knowing the profile of falls, regarding its characterization and the patients’ comorbidities^7^ ^-^ ^8^, it is important to know the outcomes in occurrences with moderate to severe damage^8^. The relevance of this event is perceived by the amplitude of its report to the National Health Surveillance Agency, which can have repercussions on the image of the institution, with a great chance of triggering guilt in the workers who, therefore, can be characterized as second victims in AE^9^.

Although an AE with severe outcome and even death brings suffering to the patient and their families, they are the first victims of this process, but not the only ones. The professionals involved, directly or indirectly, also suffer, being identified as potential second victims, especially when there is no institutional emphasis on well-being and systemic focus^10^ ^-^ ^12^. Feelings such as fear and guilt for the patient’s outcome are part of the second victim’s accounts^13^ ^-^ ^15^.

International Studies^12^ ^,^ ^14^ ^,^ ^16^ have already signaled the repercussions of AE on second victims; however, the Brazilian context needs to be explored regarding this issue. This study is anchored on the relevance of falls in the global context of patient safety, the gap in knowledge about the perspective of the worker facing this scenario, using process modeling, the latter two being the differential of the studies published so far. The study aimed to analyze the falls of adult hospitalized patients and their repercussions on the Nursing worker as the second victim.

## Method 

### Type of study

This is an exploratory, descriptive study with a qualitative approach, supported by Content Analysis. The study was conducted in two sequential stages in order to achieve the proposed objective and its presentation followed the guidelines of the Consolidated Criteria for Reporting Qualitative Research (COREQ).

### First stage of the study

*Location*

The study was developed in a general university hospital, reference for high complexity, with capacity for 843 beds, in the South region of Brazil.

*Period*

Data collection was conducted in the period August and September 2019. 

*Population*

Adult patients, hospitalized between July 2018 and July 2019, who experienced falls (N= 447), in adult inpatient units (N=242).

*Selection criteria*

Adult patients admitted to clinical and surgical inpatient units who had a fall inside the institution and had an injury grade ranging from two to four (moderate to death). Regarding the injury severity classification^8^ ^,^ ^17^ ^-^ ^18^, the institution of the study classifies as: (zero) no harm; (one) mild harm, one that involves minimal or moderate repercussion, but with rapid duration, requiring few interventions; (two) moderate harm, with minimal need for intervention, increased length of stay, harm or loss of function in a long term; (three) severe harm, one that has severe symptoms, with need for life support intervention or major clinical/surgical intervention, permanent or long-term loss of function and (four) death.

Exclusion criteria were considered to be patients with falls without damage or with mild damage (as these are considered to have less repercussion for the worker).

*Sample*

The sample consisted of 12 falls, which consisted of the total number of falls with moderate to severe damage recorded between July 2018 and July 2019.

*Data collection*

Data collection was performed in the institutional database by means of the Management Information System (falls notified in the computerized system), Strategic and Operational Management System (Standard Operating Procedure - SOP and Protocol for Prevention of Falls in Adults) and information from medical records. 

*Data analysis*

The data was entered into the FRAM Model Visualiser Software^*^ and their analysis occurred in two stages: initially, the data referring to the prescribed process (SOP and Protocol for Prevention of Falls in Adults) and, afterwards, the data referring to the real work (management information and medical records related to falls). The software used modeled (drew) each process (prescribed and real) presenting, as a result, a graphic illustration.

### Second Step of the Study

*Period*

The data collection was carried out in the period from March to May 2020.

*Population*

The population consisted of nurses and nursing technicians working in clinical and surgical admission units, consisting of a total of 265 professionals.

*Selection criteria*

Nurses and Nursing technicians allocated in one of the inpatient units where falls with moderate severity of injury to death occurred were included (according to the results of the first stage of the study). Exclusion criteria were: professionals who worked for less than a year in the institution, professionals on vacation or leave of any kind, besides those on temporary contract during the data collection period.

*Sample*

The sample was defined by simple random drawing. One nurse and two Nursing technicians were chosen for each shift in the units where the falls occurred. Twenty-one professionals were selected, following the data saturation criterion, considering the proportional distribution to the five inpatient units selected in the first stage of the study and the number of professionals in each category (nurses and nursing technicians). Among those chosen, one professional declined the invitation and another, due to problems in the unit, was unable to participate.

*Data collection*

Semi-structured interviews were conducted with a script based on the study objective and the results of the first stage. The interviews took place during the participant’s work shift, with an average duration of 35 minutes, were audio recorded and later transcribed. 

The interview script was prepared by the researchers based on the literature. At first, a pilot interview was conducted with a professional who did not belong to the units included in the study as a way to calibrate the data and adapt the interview script. The questions that guided the interviews were: how do you receive information about the falls that occur in your unit? In your understanding, why do patients fall? When considering the institutional results (presented in the FRAM modeling in the actual work process), what do you think could be done to optimize fall prevention? When a fall with patient harm occurs, do you believe there are repercussions for the professionals involved? Tell us about a fall experienced by a patient under your responsibility or of a colleague in the sector, the repercussions for the patient and for the worker, as well as the approach taken with the professional involved.

The interviews were conducted by the master’s student, the proponent of the study, who is a nurse and has experience in conducting semi-structured interviews and periodically attended meetings with the research team. All interviews were conducted face-to-face, in a shift and time of preference of the participant. The interviews took place inside the professional’s work unit, in a reserved place.

The professionals who agreed to participate in the interviews were provided with information about the institutional data and the theme of the study^**^.

*Data analysis*

The Content Analysis technique was used according to Bardin^19^, following the three-stage systematics: (i) pre-analysis, (ii) exploration of the material, and (iii) treatment of results, inference and interpretation. The first stage consisted of the organization of the material, floating reading and exhaustive reading of the interviews that made up the corpus of the research. In the second stage, the coding units were chosen and the categories were formed by classification and aggregation. In the third stage, inferences and interpretations were made in order to make the data meaningful and valid.

*Ethical aspects*

This research complies with Resolution No. 466, 2012, of the National Health Council. This is a project linked to an investigation previously approved by the Research Ethics Committee of the institution in which the study was conducted under number 2,554,758.

For the use of the hospital databases, a Term of Commitment for the Use of Institutional Data was filled out, and the participants of the interview were given an Informed Consent Form. The participation of the workers in the study was voluntary, with no implication on their employment relationship and work relations. In order to maintain the anonymity of the participants, the workers’ statements were coded NT for nursing technicians and NUR for nurses, followed by the numbering referring to the order of data collection.

## Results

### Related to the second step of the study

*Occurrence of falls in patients with moderate to severe damage*

Falls during the period July 1, 2018 to July 31, 2019 totaled 447 occurrences, with falls in adult inpatient units being 242 (54.1%) and 12 (2.7%) representing falls with moderate to severe harm occurring in five clinical and surgical inpatient units.

*Process Modeling in Falls of Hospitalized Adult Patients*

The functions (represented by a hexagon) were modeled in the moments recommended for the application of the scale according to the Protocol for Prevention of Falls in Adults, in which the risk is identified by means of a stratified score. The patient identified as being at risk of falling receives a yellow bracelet, which provides visual signaling to the professionals. Next, the functions that concern the record in medical records and the guidelines that are given to the Nursing team were modeled, more specifically, to the Nursing technician responsible for implementing the care to the patient (and his/her caregiver or companion) in that shift.

**Figure 1:**
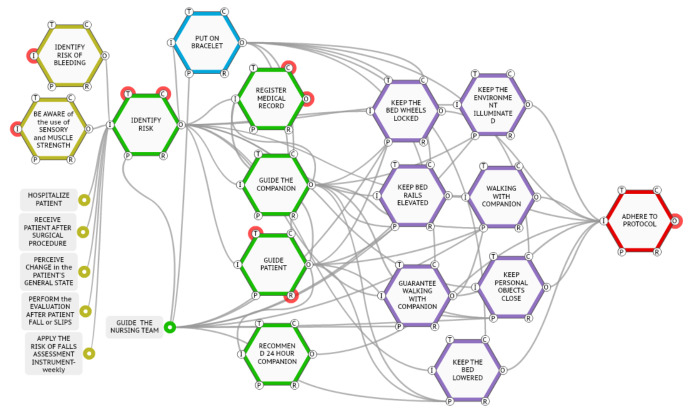
Graphic illustration of the prescribed process modeling for falls prevention in hospitalized patients. Porto Alegre, RS, Brazil, 2019

The modeling sequence also allowed the identification of the care that is oriented and the adherence to all these orientations, both by the Nursing team and by the patient, his/her family member or caregiver, aiming at the non-occurrence of the fall. The functions listed for the prescribed work are presented in Figure 1.

In the vertices of the hexagons (functions), the letters I, P, R, T, C and O stand for: Input, Precondition, Resources, Time, Control and Output, respectively. 

After modeling the prescribed process, the 12 falls with moderate to severe damage were analyzed, nine of them occurring during the night shift. The detailed analysis of each one enabled the grouping by variability of function and, after that, the union by similarity of variability, composing the analyzed cases presented in Figure 2.

**Figure 2:** Grouping of falls by similarity of variability, composing the cases. Porto Alegre, RS, Brazil, 2019

Case	Variability
A (Falls 1 and 11)	Pay attention to the use of drugs that alter sensory, muscle strength; Noticing changes in the patient’s general condition; Instruct a companion; Recommend a companion; Ensure accompanied ambulation
B (Falls 2, 3 and 12)	Perceiving a change in the patient’s general condition; Ensure accompanied ambulation; Identify the risk
C (Falls 4,5,6,7,8 and 9)	Perceiving a change in the patient’s general condition; Ensure accompanied ambulation; Identify the risk
D (Fall 10)	Identify the risk; Identify the risk of bleeding; Putting on a bracelet; Pay attention to the use of drugs that alter sensory and muscle strength; Guidance to companion; Guiding the patient; Guide the nursing team

**Figure 3:**
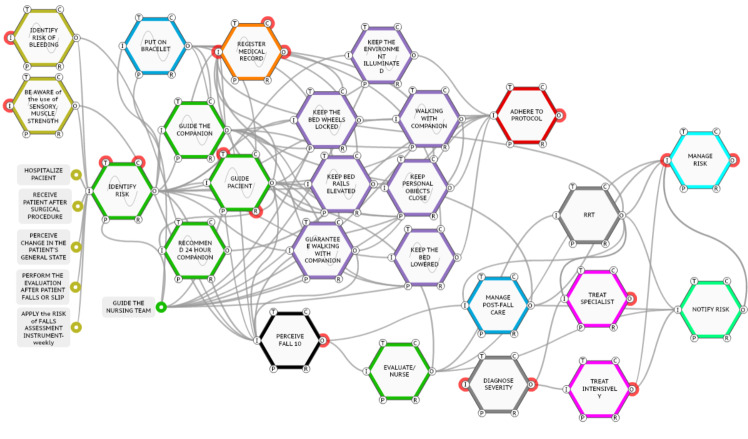
Graphic illustration of the actual process modeling of Case D. Porto Alegre, RS, Brazil, 2019

The modeled fall 10 was used to support the interview in the second stage of the study (Figure 3).

*Falls from the perspective of the nursing worker*

Two categories allowed us to understand the implications of falls for nursing workers.

The findings related to the interviews formed the categories (1) The complexity of care to prevent falls (composed by the subcategories “One protocol, many cares” and “Multi-professional dimension of the fall”) and (2) Feelings of the second victim (originated by the subcategories “Suffering and guilt” and “Concern that lasts”) presented below.

*The complexity of care to prevent falls*

The complexity of fall prevention is demonstrated in the institutional protocol and in the multiplicity of professionals who interact with the patient. The institutional protocol for the prevention of falls indicates that professionals provide guidance to patients and families, among other care in which the Nursing team is supported by.

*(...) I took care of the railing, I oriented the family members that they should not pick up the patient, because I left everything close enough that he doesn’t need to stretch himself (...).* (NT1)

*(...) make sure that he has a bracelet, that the bed is lowered, that the bars are raised.* (NT3)

The hospital environment, given its complexity, requires planning and organization so that the activities are executed in a coordinated manner, aiming to achieve, among other objectives, patient safety. However, it is possible to notice that the prescribed work is relativized by the human condition present in the interactions of the complex environment that is the hospital.

*(...) patients have a lot of resistance, (...) I believe that they feel trapped, with their freedom limited. (...) we, the nursing staff, we end up giving in, and (...) favoring that the patient ends up falling (...).* (NUR2)

Fall prevention involves multi-professional dimensions, by means of a shared look focused on the patient and considering that there is always the risk of falling.

*(...) the cleaning lady left the trash in the hallway, the patient gets there and thinks that he is going to deviate, and there by the corner of the hallway, (...), but xxx was taking care of the patient, didn’t he see this? And wouldn’t it help, wouldn’t it have a better result, if the cleaning lady collected the garbage? Before, the girls from the kitchen put the diet on the patient’s bedside and rang the bell, (...) in terms of the multi-professional team, if there was a participation of the whole team, things would be easier.* (NT1)

With the purpose of a multi-professional look, the interviewees point out that falls need to be seen from the perspective of the different actors that interact in the scenario, in order to strengthen the multi-professional and inter-professional aspects of fall prevention.

*(...) when the patient falls, it is our responsibility, it is the responsibility of Nursing (...).* (NUR7)

*(...) it seems like a tug-of-war (...) we are not helping each other at all (...). There is a lack of a multi evaluation of this patient, interdisciplinary, that we realize what we have to do. (...) this is because we see a cycle of disagreements (...).* (NUR6)

Fall prevention requires recognizing the need to engage the entire multi-professional team in order to share responsibilities.

*Second victim’s feelings*

The findings show negative feelings linked to the experience of the fall.

*(...) anguish, a fear, (...) but also in relation to our professional life, how much this affects people’s view of our technical capacity, in short (...).* (NUR6)

*(...) blaming yourself, (...) in practice, there was nothing to do, you get very upset... You spend days talking about it, crying for the patient, for fear that the boss will demand it (...).* (NT7)

When there are no institutional measures in place to support the worker and thus the feelings persist with the worker.

*(...) this will influence the moment I go downstairs, take my shower and get out of the gate there. (...). Then, I go home thinking about it (...), I sleep and turn on the phone (...) it will stay in my mind.* (NT10)

The incipient approach to the feelings aroused in the professionals has repercussions on the workers’ health. At times, workers question the relationship of trust with the patient, understanding that they can become victims if the blame becomes explicit and the reason for even more demands.

*(...) the nursing technician is also a victim (...) I’ve seen family members wanting to get money on top of this, pressuring the boss to fire them, humiliating us to get off the scale (...). (NT1)*

When the guidelines are not followed, the worker does not recognize his effort for the appreciation of the other, that is, the usefulness of the actions is not recognized by the patient, generating negative feelings that add to the own guilt for the fall.

*(...)”Lady, he’s going to slide!”. That was it, right? It seems that they don’t trust what we say, (...) they think that we want to tie them up (...) so that they don’t have to work (...).* (NT4)

*(...)At the same time, a feeling of injustice. If people were here watching, they would see that there are things that are out of our control (...).* (NUR7)

The analysis of an adverse event requires a look at the professionals involved, as a way to understand the difficulties that go beyond the prescribed work, since the adversities involved in trying to perform the work previously planned provoke feelings that are sometimes ambiguous.

*Regardless of anything, if he falls, it is my responsibility, he was under my care. But the system doesn’t allow me to stay there by his side (...).* (NT13)

The feelings that the patient awakens in the team reflect in a new configuration that must be made, in haste, to accommodate the demands, repercussions in a greater workload and even in the relationships of trust previously established between the teams, but that need to meet the demands of some family members.

*I had to, by requirement of the family member, ask for a change [of scale] because of inadequate care (...).* (NUR1)

Still, the relationship between the orientation given by the Nursing team and the patient’s non-adherence may lead to the feeling that the orientations and other fall prevention measures were trivialized by the patient.

*(...)the demand and dynamics of our work is so fast paced that (...) this attention to some fall measures... lose a little attention (...).* (NUR7)

The reflection about the etiology of the fall, the individualized look, helps in the identification of risk and, consequently, in barriers and in the proposition of systemic treatment. The automation of both the prescribed care and the care provided predisposes to a feeling of indifference.

*The technician does the same function several times (...) and this gets into the memory (...). Then (...) only if it is something different (...), but the description of the fall we do not enter (...).* (NT7)

*The nursing prescription has some repetitive data, right, and this, in my point of view, is kind of a disincentive for the technician to read all that.* (NUR3)

The trivialization also happens when the support that the institution should provide does not happen in an effective way. Situations like this one lead to the devaluation of the repercussion of the event and of one’s own feelings.

*There are so many other attributions that they will not want to give psychological support to us because of a fall, I don’t see that.* (NT11)

The feeling of devaluation was also noted when the importance of support measures for the worker who was involved in a fall event was mentioned, which can be considered an addition to the condition of second victim.

## Discussion

The analysis of the modeled falls allowed the identification of the variability of the functions represented^5^ ^,^ ^20^ by the absence of the companion/family member, in patients under the effect of sleep-inducing, hypotensive medication or that provoke alterations in muscular strength. In addition, in the presence of a change in the patient’s general state, by the impossibility of the Nursing team to accompany the patient during his displacements, as well as by a need for investment, either in the patient and his family member, or in the teams that manage the care and conduction of this patient. These are facts evidenced by the professionals when they identify the importance of the qualification of the teams, of the education of patients and companions, making them partners in care^21^. It is from this perspective that resilient systems are needed as a way to reduce this variability and provide greater support to professionals.

The difficulty in contemplating the multiplicity of care revealed by the modeling of the process and by the experiences of the professionals is corroborated by the identification of a Nursing prescription with repetitive data, generating a discouragement in the Nursing technician, as well as in his speech that, by performing the same function several times, ends up not giving real importance to the fall. The prescribed care needs to reflect not only the patients’ needs, but also to allow for its implementation. Therefore, it is necessary to consider this distance with repercussion on the variability for the occurrence of falls. The patient’s prescription needs to involve all the actors who take care of him, ensuring a greater imbrication, considering the patient and his interrelationship with all parts^22^.

Regarding the time when the falls occurred, the night shift was preferentially the shift that presented the most occurrences, corroborating a previous study carried out in the same institution in which the falls of adult hospitalized patients were characterized^7^. There is a reduction in the number of professionals during the night shift and a lower circulation of people in the hospital environment, which may be related to a lower surveillance capacity and a reduction in the frequency of approaches with patients, even to not interfere with sleep, contributing to a higher number of falls in this turn. Another important point is the darkness of the room, which can increase the risk of falls, a fact that is contemplated in the Nursing prescription when orienting the maintenance of a lit environment^23^.

The implementation of collaborative practices qualifies health services to the extent that there is understanding of the dimension of the work involved, resulting in better outcomes, patient safety, and promoting relationships of trust among professionals. In this context, this collaborative practice must reflect the joint construction and, therefore, it is necessary to rethink the activity performed, in addition to criticize and act, but to think, plan and replan the work focused on the care that will be offered to the patient, investing in the multidisciplinary team, aiming to reduce falls, an occurrence that interferes with the continuity of care and patient safety^21^ ^-^ ^22^ ^,^ ^24^.

Although, as a profession, there is a need to follow rules and SOP, these must be of joint construction, considering that, in the face of unexpected situations, which the SOP cannot contemplate, the solutions must emanate from the teams.

The scale that assesses the risk of falling is applied by the nurse, however, several other professionals, not only Nursing, orbit around this patient and, more than that, one can compare that the patient is in the center of a discussion, such as a tug-of-war, in a cycle of disagreements among professionals. This is understood as a clear and elucidative demonstration of the lack of cohesion among professionals, between the prescription and the professionals, exposing them and the institution. This is a strong inference that is made about the feeling of guilt, anguish, impotence, of not having cared enough, of devaluation for the lack of adherence of the patient and his family/caregiver.

In hospitals, complex environments^3^, there is an interaction between different professionals, who orbit around the patient, with different levels of performance, including adding technology to care. In this context, the importance of teamwork is emphasized, which, unlike compartmentalized work, enhances care, through joint reflection, bringing benefits to the teams and the patients^24^ and broadening the interaction among people, in the joint search for better results.

In this sense, contrary to the potentiality of teamwork, the SOP and the protocol for falls prevention are seen as items to be fulfilled by Nursing. Therefore, when something does not turn out as expected, Nursing projects this negative outcome onto itself, because this work did not happen in a joint manner, added to the fact that there is great difficulty in accepting the fallibility of the profession^25^.

Although the feeling of being close to a patient who suffered a severe injury is something mobilizing, as brought up by the professionals of feeling guilty, of crying for the patient, of going home and thinking about what happened, and even, in the judgment, of being labeled as incompetent, other feelings are raised, such as trivialization. The literature, even though in its definition of harm it alludes to the patient, makes it possible to appropriate the concept and apply it to the second victim when it includes the repercussion on suffering, which can have social or psychological repercussions^26^.

Moreover, in the context analyzed, in which the number of events with moderate to severe damage represents 2.7% of the occurrences, one has the false impression that this percentage is not very expressive. However, it reflects that a very large extract of events without damage and with mild damage occur in the same way as the moderate and severe ones, but these are not treated, thus allowing little appreciation, or even trivialization of situations with unfavorable outcomes.

Nursing is a profession strongly rooted in the final result, disregarding the fallibility of what is performed, increased by the complexity of the activities. The tolerance to errors, on the part of the institution, helps in the behavior of the professionals, having repercussions in the mediation of the learning process, in psychological security, because the hospital institutions seek perfection, projecting attitudes towards error^15^ ^,^ ^23^, can foster feelings in the second victim.

Among the feelings, guilt for the patient’s outcome, fear for the loss of reputation and anguish for the situation not being addressed are part of the second victim’s experiences^15^ ^,^ ^26^, and Nursing, especially, being part of the front line, is more susceptible to being a second victim^16^.

The feelings of the second victim provide negative reactions. Among the feelings reported, guilt, stress due to the concern for the patient, fear of judgment and the outcome for the patient elucidate what is also brought up in the international literature^11^ ^,^ ^13^ ^-^ ^15^. These are common feelings in the face of error, as it demonstrates human fallibility and the consequent emotional impact, with increased absenteeism and intention to quit^11^. Spending days thinking about what happened, crying for the patient and suffering with the family are part of the professionals’ daily practice. These repercussions are directly related to the degree of the patient’s unfavorable outcome^15^ and that, in a certain way, are corroborated by the chief’s judgment when he changes a professional’s scale at the request of a family member or when he doesn’t receive support from his colleagues, given the volume of work that continues to be demanded, reiterating the need for a support infrastructure for the second victims^10^ ^-^ ^11^ ^,^ ^13^ ^,^ ^16^.

The support to second victims of events with damage to patients is valuable for coping, and it ranges from participation in groups for analysis of events, as a form of a systemic look at the processes, to even encouraging the sharing of experiences as a second victim, constituting a restorative justice. And, as a way to work fomenting a fair culture, it is important that the workers are encouraged to recognize the possible contribution in the damage to the patient, reinforcing the measures for the treatment of both the first and second victims^27^ ^-^ ^28^. Strategies of emotional support to the second victim and qualification of work processes require commitment from leaders^10^. Leadership best practices impact hospital risk management^29^, on the safety climate and staff satisfaction^30^, through early worker support.

Since this is a small-scale study, the results are sensitive to the context employed, which is a limitation. However, this methodology can be replicated, allowing investment opportunities to be identified, as in the case of training approaches for professionals and educational approaches for patients, companions and families.

Although with a strong contribution from the educational point of view, the identification of work processes built in a multiprofessional way is, without a doubt, an important contribution of the study. The approach to falls needs a systemic, two-way look, i.e., think about the structural, protocol context, but not in a rigid way that disregards the social context, the experience of patients and their companions, of professionals and the knowledge from previous experiences.

## Conclusion

This study identified 447 falls of adult hospitalized patients, 12 events with moderate to severe damage, corresponding to 2.7% of the notifications with this profile, which occurred predominantly at night. The variability of the event occurred in the absence of a companion/family member, in patients under the effect of sleep inducing drugs, hypotensive drugs, or drugs that cause alterations in muscle strength. In addition, in the presence of a change in the general condition of the patient, due to the impossibility of the Nursing team to accompany the patient during his displacements.

The understanding of the multimodality of falls was possible to the extent that Nursing professionals identified, through the modeled falls, situations that compromise the quality of care and whose prevention requires the involvement of the patient, family, and multi-professional team. The professionals involved are second victims and experience feelings of guilt, fear, anguish, impotence and even trivialization when facing events that have repercussions for the workers.
